# Gingival Phenotype Changes and the Prevalence of Mucogingival Deformities during the Early Transitional Dentition Phase—A Two-Year Longitudinal Study

**DOI:** 10.3390/ijerph19073899

**Published:** 2022-03-25

**Authors:** Agnieszka Kus-Bartoszek, Mariusz Lipski, Anna Jarząbek, Joanna Manowiec, Agnieszka Droździk

**Affiliations:** 1 Independent Laboratory of Paediatric Dentistry, Faculty of Medicine and Dentistry, Pomeranian Medical University in Szczecin, 70-111 Szczecin, Poland; kusia33@poczta.onet.pl (A.K.-B.); anna.jarzabek@pum.edu.pl (A.J.); joanna.manowiec@pum.edu.pl (J.M.); 2 Department of Preclinical Conservative Dentistry and Preclinical Endodontics, Pomeranian Medical University in Szczecin, 70-111 Szczecin, Poland; mariusz.lipski@pum.edu.pl; 3 Department of Interdisciplinary Dentistry, Pomeranian Medical University in Szczecin, 70-111 Szczecin, Poland

**Keywords:** gingival phenotype, gingival thickness, gingival width, mucogingival deformities

## Abstract

Thin gingival phenotype (GPh) may contribute to periodontal tissue breakdown and recession development. Thus, the early identification of thin GPh in children can allow proper preventive care and the identification of children at risk during orthodontic treatment. The present long-term study aimed to monitor GPh changes, i.e., thickness (GT) and width of attached gingiva (AGW) during the early transitional dentition phase, as well as its potential associations with the mucogingival deformities. Materials and Methods: 83 systematically healthy children were examined twice with an interval of 2 years. Probing depth, GT and AGW at mandibular incisors, vestibular depth, type of lower lip frenum attachment and mucogingival defects were recorded. Results: 95.2% of participants at baseline and 93.9% at 2-year examination expressed thin GPh. During the transition from the deciduous to permanent dentition, GT and AGW declined, but the GT of permanent incisors already erupted at the baseline examination increased in the observation period. Conclusions: Gingival phenotype undergoes changes in the early transitional dentition phase. In spite of the thin gingival phenotype, only single pseudo-recessions and primary shallow vestibule were noticed.

## 1. Introduction

The diagnostic assessment of soft and hard tissues surrounding the tooth is one of the most crucial factors in clinical practice. It may influence treatment planning and the decision-making process for multidisciplinary dental treatments [1,2,3]. As various gingival/periodontal biotypes respond differently to similar chemical, physical and bacterial injuries or trauma during dental treatment, it is also considered to be an essential factor determining the outcome of therapy in many dental disciplines [4,5,6,7]. 

In clinical applications the term phenotype instead of biotype seems to be more appropriate since it may be modified by either environmental factors or clinical therapeutic interventions, including iatrogenic effects [8,9]. 

According to the 2017 World Workshop on the Classification of Periodontal and Peri-Implant Disease and Conditions, two factors were proposed for description of periodontal phenotype characteristics: (a) the gingival phenotype, which describes the morphology of the soft tissues and comprises the thickness and the width of the keratinized gingiva and (b) the bone morphotype, referring to the thickness of the buccal bone plate [6,9]. In addition to those two elements, the size and the shape of teeth are also evaluated to distinguish the phenotype [3].

Understanding gingival phenotype may be of importance in paediatric dentistry, given that thick gingiva provides strong and stable base for keeping optimal oral hygiene and is one of the essentials in orthodontic treatment [10,11].

According to recent research, a minimum amount of keratinized tissue is not needed to prevent attachment loss when optimal plaque control is maintained in natural teeth. However, it has been demonstrated that the narrow zone of keratinized tissue in the presence of a dental biofilm favours the apical migration of marginal gingiva, especially in teeth undergoing orthodontic treatment [3,12]. Thickness of the gingiva in bucco-lingual dimensions, a second parameter characterizing gingival phenotype, in combination with direction of the tooth movement are considered important in soft tissue alteration and development or progression of mucogingival defect [1,6,9,13]. In case of a thin phenotype, dehiscence and/or fenestration, predisposing to gingival recession may occur if the tooth is moved beyond biological limitations, i.e., outside the osseous envelope [3,12,14,15,16]. The mandibular incisors were found to be the most susceptible teeth to labial recession development [11,17]. Some malocclusions become evident during the early mixed dentition period; on the other hand, this phase offers a great opportunity for the implementation of interceptive orthodontics [18].

There are intense changes in the stomatognatic system in children during the developmental period, including the alteration in the mucogingival complex and periodontium. Many of those changes are genetically determined, others result from biological environmental factors, for example tooth position in the arch [19,20]. As in early childhood mucogingival deformities may develop with a resultant impact on permanent dentition [21], clinicians should bring awareness to their prevention and careful monitoring.

In the past decade, the periodontal biotype/phenotype was investigated in numerous studies, the majority of them included the upper anterior teeth. There are only limited data available on the correlation of the thickness and the width of the keratinized tissue with other teeth, including lower incisors [20,22,23,24]. Moreover, those studies focused on the adult population. 

In light of the above, the present two-year longitudinal study was undertaken to obtain information on the developmental changes in the gingival phenotype during the early transitional dentition phase in children, and to correlate them with mucogingival deformities. The objective of the study was to assess GT and AGW changes at mandibular incisors in the study period.

## 2. Materials and Methods

This prospective longitudinal study was performed in accordance with the Helsinki Declaration of 1975, as revised in 2000, and the protocol was approved by the local ethics committee (KB-0012/72/17). The oral consent from all children, preceded by the written informed consent from parents/caregivers was obtained. 

### 2.1. Study Population

**Figure 1:**
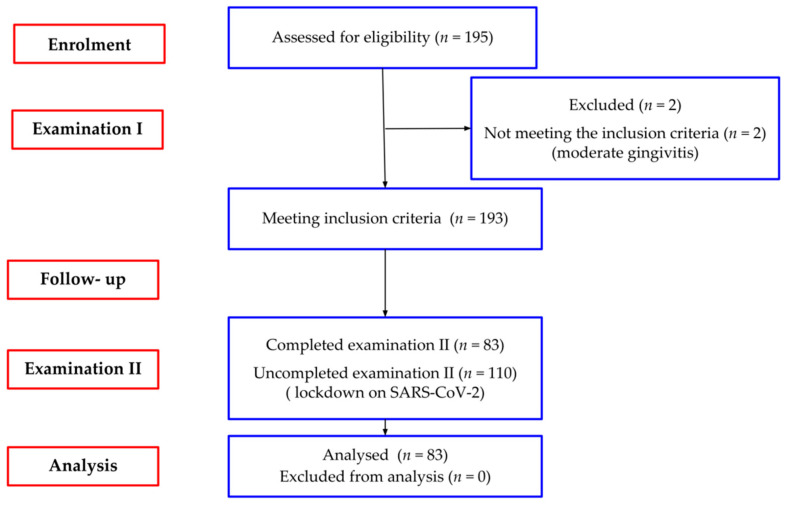
Study flow chart (*n*—number of participants).

Enrolment and baseline examination was conducted in four randomly selected primary schools from January 2018 to May 2018. One hundred and ninety-three (out of 195 examined) seven-year-old children (92 girls and 101 boys) at the age of 72–97 months (mean 86.28 ± 4.5, Me 86) met the eligibility criteria (Figure 1). 

The inclusion criteria were as follows: mandibular incisors fully (incisal edge reaches occlusal level) or partially (no less than 50%) erupted, no clinical attachment loss, healthy gingiva or mild gingivitis (gingival index, GI ≤ 1). Excluded were individuals with systemic diseases and medications affecting gingival tissues. The subjects were re-examined after two years, from the entry evaluation. Due to the SARS-CoV-2 pandemic, 2-year-examination was possible in 83 children (41 girls and 42 boys), and those children formed the study group (mean age 87.11 ± 4.5-month, range 72–97, Me 88).

A total of 287 lower incisors, including 159 central and 128 lateral, were assessed at baseline examination, and among them 56 incisors were deciduous, 201 permanent partially erupted and 30 fully erupted. At the examination II, 332 lower incisors were explored, all were permanent, 236 of them were classified as fully erupted, 96 as partially. The number of lower central and lateral incisors was the same (*n* = 166).

### 2.2. Clinical Examination

The study participants were evaluated in two clinical examination sessions by an experienced and prior calibrated examiner (A.K.-B.). Five paediatric patients, not included in the study, as reported for caries treatment in the laboratory, were used for calibration. The examiner evaluated probing depth (PD), keratinized tissue width (KTW), vestibule depth (VD) at lower incisors at each patient on two separate occasions of one week interval. The calibration was accepted if the measurements at the baseline and one week apart were equal to the half of millimetre at more than 90% level.

The following parameters were assessed at the lower incisors:

- Plaque Index (Pl. I)—site specific evaluation of the presence of dental plaque according to the Silness i Löe criteria [25];
- Gingival Index (GI)—site specific assessment of the condition of the gingiva according to the Löe i Silness criteria [26];
- probing depth (PD)—at the mid-buccal aspect of the examined tooth from the gingival margin to the bottom of the gingival sulcus;
- keratinized tissue width (KTW)—at the mid-buccal aspect of the examined tooth from the gingival margin to the mucogingival junction using wrinkle technique [27];
- attached gingiva width (AGW)—subtracting the PD from the KTW;
- vestibule depth (VD—at the central lower incisor, from the gingival margin to the greatest concavity of the mucolabial fold reduced by the PD.

PD, KTW and VD were measured with the use of the University of Michigan “0” probe (Hu-Friedy, Chicago, IL, USA) with Williams markings and were rounded to the nearest 0.5 mm.

- thickness of attached gingiva (GT) was measured by PIROP ultrasonic biometer (Echo-Son, Puławy, Poland) with a fine transducer head (1.7 mm) (device frequency-20 MHz, ultrasonic impulse velocity-1540 m/s, accuracy up to 0.01 mm) [28]. The measurement at each point (halfway between muco-gingival junction and free gingival groove) was taken twice. The final recorded value was the arithmetic mean of 10 correctly performed measurements, automatically calculated by the device. In the case of a difference in the measurements greater than 0.05 mm, a third measurement was made. The mean value of the obtained measurements was subjected to analyses.
- presence of mucogingival deformities, such as gingival recessions, lack of keratinized gingiva, shallow vestibule, abnormal frenal attachment, gingival enlargement.

Frenum position assessment was performed using Placek classification [29]:

1. mucosal—the frenal fibers attachment is positioned at the mucogingival junction;
2. gingival—the frenal fibers attachment is inserted within the attached gingiva;
3. papillary—the frenal attachment extends up to the interdental papilla;
4. papilla penetrating—the frenal attachment crosses the alveolar process and extends up to the palatine papilla.

### 2.3. Statistical Analysis

All statistical analyses were carried out using R packages, 4.0.2. edition, R Foundation for Statistical Computing, Vienna, Austria, R: A language and environment for statistical computing. URL https://www.R-project.org/ accessed on 25 June 2021. In statistical descriptions mean, standard deviation and median were calculated. The comparison of quantitative variables in the two repeated measurements was performed with the Wilcoxon matched pairs test. The analysis of changes in two repeated measurements for qualitative variables was performed using the McNemar test (for binary categorical variables) or the Bhapkar test (for multiple categorical variables). The threshold for statistical significance was set at *p* < 0.05.

## 3. Results

### 3.1. The Hygiene and the Gingiva

At the baseline and at the 2-year examination, oral hygiene in incisors segment was comparable, and the value of Pl. I indicated good hygiene. The Gingival Index values were different, at the 2-year examination GI was significantly lower (Table 1).

### 3.2. Probing Depth

At the first and the second examination, the smallest recorded depth was 0.5 mm, the greatest at the baseline examination was 6 mm, and in the second–3 mm. During the two-year observation period, the mean PD was significantly reduced by 0.22 mm (Table 1). The analysis of PD changes including the type of incisors (central/lateral) showed a significant reduction in PD only within the central incisors (by 0.33 mm) (Table 1).

### 3.3. Gingival Phenotype

#### 3.3.1. Gingival Thickness

Thin GPh (gingival thickness ≤ 1 mm) was definitely the dominant phenotype observed among the studied group. At the first examination, 79 participants out of 83 (95.2%) expressed thin GPh, whereas during the re-examination 78 individuals (93.9%) expressed thin GPh. 

The thinnest gingiva at the baseline examination was only 0.29 mm thick, the thickest 3.18 mm, and at the second examination 0.29 mm and 1.58 mm, respectively.

The observation revealed a significant reduction (by 0.03 mm) in the average GT in the two-year early transitional dentition phase (Table 1). GT presented a different tendency, when account was taken into the type of incisors (central/lateral). In central incisors the attached gingiva became significantly thicker (by 0.04 mm), in laterals thinner, but not statistically significant.

**Figure 2:**
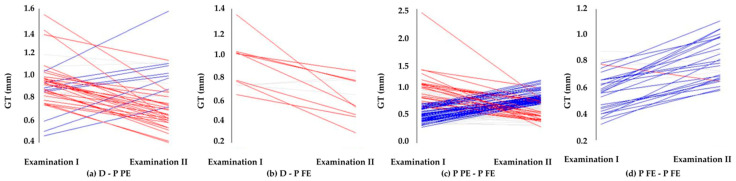
Gingival thickness (GT) changes noticed after (**a**) the replacement of a deciduous tooth (D) with a permanent one partially erupted (P PE), (**b**) after the replacement of a deciduous tooth (D) with a permanent one fully erupted (P FE), (**c**) during eruption (P PE–P FE) and (**d**) after complete eruption (P FE–P FE).

The analyses of GT changes in relation to the tooth type exchange, i.e., deciduous incisor (D) to partially (P PE) or completely erupted permanent incisor (P FE) as well as the degree of eruption (P PE-P FE and P FE-P FE) were also carried out. Transition D with permanent successors, independently partially (D-P FE) or completely (D-P FE) erupted, was accompanied by a statistically significant reduction in GT (Table 2). The GT reduction associated with the replacement of the D-P FE was almost twice higher than the D-P PE, by 0.27 vs. 0.14 mm, respectively. In contrast, the average GT of the lower permanent incisor already presented during the first examination increased significantly by 0.07 mm, while continuing the eruption (P PE-P FE and P FE-P FE). A particularly greater increase in GT (by 0.21 mm) was noted in the area of the P-FE at the baseline examination. GT changes are graphically shown in Figure 2.

#### 3.3.2. Width of Attached Gingiva

The range of measurements of the AGW at the baseline examination was from 0 to 6.5 mm, and at 2-year examination from 0 to 6.0 mm. A lack of an attached gingiva was observed in two participants at baseline examination and at re-examination.

During the two-year observation period, AGW decreased statistically significantly by 0.63 mm (Table 1), both in the area of the central and lateral incisors. However, a greater reduction was observed in the central incisors by 0.71 vs. 0.57 mm, respectively. 

The analysis of AGW was performed in the same way as for the thickness, with consideration of a tooth type replacement and degree of eruption. The average AGW decreased statistically significantly after the exchange of the incisors (in D–P PE by 0.83 mm, in D–P FE by1.54 mm) and during the eruption process (P PE–P FE by 0.64 mm). However, there was no significant difference in AGW in the area of the permanent incisors that were fully erupted while the first examination (Table 3). 

### 3.4. Mucogingival Deformities

At both examinations no gingival recessions and gingiva or papilla enlargement were noticed. The information about the lack of attached gingiva is presented above (Section 3.3.2).

#### 3.4.1. Pseudo-Recessions Appearance

**Figure 3:**
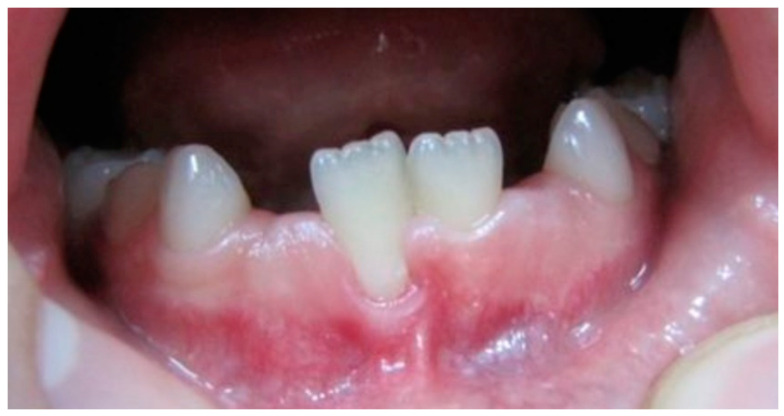
Pseudo-recession, lack of attached gingiva.

At the first examination, one boy presented a pseudo-recession (Figure 3) that was not observed at the re-examination. The 2-year examination found three new pseudo-recessions, that similar to the first examination were localised at central incisors.

#### 3.4.2. Vestibule Depth

At the first examination VD ranged from 2.5 to 13 mm, at the re-examination, two years later from 4 to 11 mm. The mean VD did not differ significantly at both examinations (Table 1). The first examination revealed three participants with shallow (≤4 mm) vestibule, at re-examination two from these measurements increased to 6 and 7 mm, one measurement did not change. At the 2-year examination two other children expressed shallow vestibule.

#### 3.4.3. Frenal Attachments

**Figure 4:**
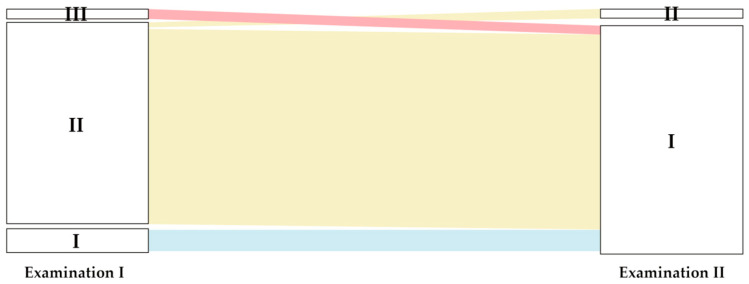
Change in the position of the lower lip frenal attachment, I—mucosal attachment, II—gingival attachment, III—papillary attachment.

The most frequently observed type of lower lip frenum attachment at the baseline study was the gingival attachment (II) (*n* = 73.88% participants). Eight children (9.6%) expressed the mucosal attachment (I) and 2 (2.4%) the papillary attachment (III). The gingival attachment, diagnosed at the first examination, after two years turned into the mucosal attachment in 96% of cases (*p* < 0.001) (Figure 4). This was the dominant type at the second examination (*n* = 81, 97.5% of cases). The papillary attachment (III) at the baseline examination evolved to the mucosal type (I) during the two-year growth up period. Papillary penetrating attachment (IV) was not observed.

## 4. Discussion

Gingival phenotype assessment should be a constant part of dental examination in patients requiring dental treatment, including children. Early diagnosis of thin gingiva, which increases the risk of periodontal problems, allows to implement preventive measures. Careful phenotype evaluation should precede implementation of orthodontic therapy, as on its basis it is possible to identify children at risk of gingival recession, which is always difficult to treat at the young age. 

There are a lot of data in the literature on the periodontal phenotype, unfortunately only a few are dedicated to children [30,31]. Furthermore, no studies reporting long-term changes of GPh and their relationship with mucogingival deformities during developmental period (which was addressed in this study) have been found. The investigation focused on mandibular incisors, which are teeth of great concern regarding recession development, especially after a change of tooth inclination [3] as well as transitional from the deciduous to permanent dentition phase, being critical period requiring close supervision [30].

Various methods have been proposed to assess gingival phenotype [3]. Not all of them are suitable for children, such as CBCT which is related to exposure to radiation [3]. Direct visual assessment was the first applied method, which in many studies was proven to be unreliable [32,33]. A simple, widely used method in adults, based on transparency of periodontal probe through the gingival margin while probing [34] as well as transgingival probing carried out under local anaesthesia might be challenging in children [3,34]. Ultrasonic measurements introduced to overcome those difficulties [35] were proposed as the most adequate for direct gingival thickness measurements from all non-invasive methods [22,27,28,36,37,38]. 

Biometric measurements for the pediatric population in the presented study were selected. GT was assessed by the ultrasonic biometer, designed for routine measurement of oral mucosa thickness. The reduced transducer head diameter enabled fast, measurements well-accepted by children. In study by Kloukos et al., an ultrasound measurement was proposed as an adequate diagnostic option for every day practice out the four methods tested at the area of lower incisor (trans-gingival probing either with periodontal probe or acupuncture needle, probe transgingival translucency, ultrasound) [22]. Dridi et al. recommended gingival whitening with coronal labial traction and visibility of blood supply as the first-line diagnosis of thin gingiva in children [30]. 

In the present study most of the participants expressed a thin gingival phenotype diagnosed when GT was measured ≤1 mm according to a threshold determined by several investigators [32,38,39]. Thin gingiva in 95.2% of seven-year-old children (baseline examination) and 93.9% of nine-year-old (second examination) was observed. During the two-year observation period, the average thickness decreased from 0.76 mm to 0.73 mm. Similarly, mean GT less than 1 mm was reported by Kaya et al. (0.71 mm) and Agarwall et al. (0.94 mm) [40,41]. In contrast to our findings, Vandana and Savitha as well as Kolte et al. [19,20] revealed the average GT at lower incisors much greater than 1 mm, amounting to 1.73 mm and 1.70 mm, respectively, indicating the thick gingival phenotype. The differences may result from different ethnic populations (white and Asian) included into the studies.

The analysis of the GT changes in relation to the type of incisors and the degree of eruption showed interesting results. The significant thining of the gingiva was noticed from the deciduous to permanent dentition (D-P PE or D-P FE) (Figure 2a,b). This finding can be associated with the larger size of the permanent teeth in the bucco-lingual dimension [5] as well as the tongue pressure at the anterior segment of the mandible [42]. The gingival thickness reduction was greater when the primary predecessor was replaced by a permanent incisor completely erupted during the observation period. 

During the already on-going permanent tooth eruption process (P PE-P FE, P FE-P FE) (Figure 2c,d) the thickness of gingiva gradually increased. Noticeable thickening of the gingiva (by 0.2 mm) over the two-year observation period was found at the permanent incisors fully erupted at the baseline examination (P FE-P FE). The present findings indicate that, the transition from primary to permanent dentition could be a critical phase, in which temporary gingival thickness reduction takes place. These results cannot be compared with findings of other researchers due to the lack of long-term observations referring to the gingival thickness in children.

The width of gingiva, next clinical parameter determining the gingival phenotype, is easy to quantify in paediatric population, and for almost half a century has been of interest to researchers. There are some observational studies referring to width of gingiva [43,44]. 

In the present study AGW significantly decreased by 0.63 mm during the two-year observation period. A reduction in the AGW at anterior teeth during the transition of dentition was also noted by Abrishami and Akbarzadeh [45] and Kim et al. [46]. However, our results do not corroborate with two-years observations carried out by Andlin-Sobocki [43]. The author reported significant increase in AGW by 0.3 mm and 0.6 mm at central and lateral lower incisors in 7-year-old children, respectively. It is also worth noting, that the baseline AGW in our study were much wider, both at central (3.28 vs. 1.8 mm) and lateral lower incisors (3.40 vs. 2.0 mm).

An increase in attached gingiva width was also observed in several cross-sectional studies [42,47,48,49]. In the present study, the analysis of AGW changes in relation to the type of incisors and the degree of eruption showed the greatest decrease when deciduous incisors were replaced with permanent successors fully erupted (D-P FE). AGW reduction at permanent teeth fully erupted at baseline examination has not achieved statistical significance. 

In the presented study, the prevalence of mucogingival deformities during early transitional dentition phase was also estimated. Gingival recession, lack of keratinized gingiva, decreased vestibular depth, aberrant frenum position, different forms of gingival excess (AAP 1999, Consensus Report) were considered. Mucogingival deformities, which may occur in early childhood are mostly diagnosed when planning an orthodontic treatment. Disturbances of teeth development and eruption, distortion of maxilla or mandible, inadequate oral hygiene are usually causal factors [50]. The 2017 World Workshop on mucogingival conditions around natural dentition confirmed that thin gingival phenotype may lead to a higher risk for future recession development. Experts provided guidelines for clinicians referring to thin gingival phenotype, recommending prevention measures and careful monitoring. Thin gingival phenotype runs greater risk to epithelial damage and loss of connective tissue [6,7]. These statements are not supported by the present study observations. No true gingival recessions despite the commonly expressed thin phenotype were observed. Tenenbaum and Tenenbaum reported 8.3% prevalence of gingival recessions during development, mostly at permanent lower central incisors [51]. The fact that true recessions were not observed in our participants can be explained by good oral hygiene (Pl I = 0.7 at baseline examination and 0.76 at 2-year examination. The occasionally noticed pseudo-recessions (*n* = 1 at baseline examination and *n* = 3 at re-examination) were localised at central incisors and were surrounded by very thin gingiva (GT less than 0.7 mm). Additionally, in two cases lack of the attached gingiva was noticed. The false recession occurring at the first examination resolved during the observation period. 

Likewise, the shallow vestibule was only occasionally observed in our study. The first examination revealed three participants presenting shallow (≤4 mm) vestibule, at re-examination two from these measurements increased to 6 and 7 mm, one measurement did not change. At the 2-year examination in two other children shallow vestibule was noticed. None of these cases was associated with the complications, such as compromised hygiene due to restrict access or inflammation as a consequence [6,52]. 

The high position of the frenulum and high muscle attachment may additionally disrupt periodontal tissues functioning, cause diastema and increase the risk for developing a recession of gingiva or central interdental papilla [53]. In the present study the papillary frenum attachment was noticed only in two cases (2.4% participants) and only at the baseline examination, whereas the penetrating papillae attachment was not observed at all. The sporadic occurrence of pathological frenal attachments did not enable confirmation of a possible relationship with development of gingival or interdental papilla recessions. Noteworthy, in the 2-year observation period both cases of the papillary attachment developed into the mucosal attachment, similar to 96% of the gingival attachment observed in the first examination (Figure 4). These observations justify concept of refraining from surgical correction of aberrant frenal attachments in the transition dentition phase.

Published cross-sectional studies evaluating lower lip frenula attachments based on the same classification focused on older populations [29,53,54]. The gingival frenum attachment most often found at the baseline examination of our study was also mostly observed in the study by Divater et al. [54]. In turn, the trend observed at the 2-year examination with most cases of mucosal attachment, was also found in study by Placek et al. [29] in a population aged from 15 to 40 years. In addition, the authors stated that as many as 71.4% of cases of gingival attachment, 83.3% of the papillary attachment and 50% of the attachment penetrating the papilla demonstrated pathological changes in the papilla. The present study observations do not confirm these findings. 

## 5. Limitations

The results of this study referring to gingival phenotype and mucogingival deformities in the mandible anterior area in children could be beneficial for clinical practitioners The tooth position in the buccolingual dimension of the alveolar process as well as transitional crowding typical for the evaluated phase of development were not considered and can constitute a possible limitation of the study. Likewise, the application of a single method of the assessment of gingival phenotype, carried out by one clinician without testing the intra- and inter-examiner repeatability can be stated. Future studies might include both dental arches and all tooth types. Investigations of different factors influencing gingival phenotype might also be advisable to provide a strong foundation for multidisciplinary treatment of children. 

## 6. Conclusions

The results of this 2-year longitudinal study conducted in children clearly showed that:

- thin gingival phenotype was defined in the majority of participants in the early transitional dentition phase,
- both parameters describing gingival phenotype, i.e., thickness and width of the attached gingiva declined from the deciduous to permanent dentition,
- gradual thickening of gingiva is initiated by a permanent tooth eruption,
- only single mild mucogingival deformities, such as pseudo-recession and shallow vestibule, were observed.

## Figures and Tables

**Table 1:** Changes in the measured parameters.

Variables		Examination I		Examination II		*p* Value
		Mean ± SD	Me	Mean ± SD	Me	
Pl. I		0.70 ± 0.62	0.6	0.76 ± 0.66	0.8	*p* = 0.464
GI		0.18 ± 0.26	0	0.04 ± 0.11	0	*p* < 0.001 *
GT (mm)						
	all incisors	0.76 ± 0.36	0.7	0.73 ± 0.19	0.74	*p* = 0.02 *
	central incisors	0.67 ± 0.34	0.61	0.71 ± 0.18	0.74	*p* < 0.001 *
	lateral incisors	0.88 ± 0.35	0.86	0.76 ± 0.19	0.75	*p* = 0.064
AGW (mm)						
	all incisors	3.33 ± 1.01	3	2.7 ± 0.97	3	*p* < 0.001 *
	central incisors	3.28 ± 1.07	3	2.57 ± 0.96	3.5	*p* < 0.001 *
	lateral incisors	3.4 ± 0.93	2.5	2.83 ± 0.97	3	*p* < 0.001 *
PD (mm)						
	all incisors	1.47 ± 0.76	1	1.25 ± 0.51	1	*p* < 0.001 *
	central incisors	1.47 ± 0.69	1	1.14 ± 0.39	1	*p* < 0.001 *
	lateral incisors	1.47 ± 0.84	1	1.37 ± 0.58	1	*p* = 0.746
VD (mm)		7.76 ± 1.86	8	7.22 ± 1.47	8	*p* > 0.05

Pl I—Plaque Index, GI—Gingival Index, GT—gingival thickness, AGW—attached gingiva width, PD—probing depth, VD—vestibule depth SD—standard deviation, Me—median, * statistical significance.

**Table 2:** Thickness of the attached gingiva (GT) change.

Exchange/Eruption Degree	Examination I		Examination II		*p* Value
	Mean ± SD	Me	Mean ± SD	Me	
D-P PE	0.92 ± 0.20	0.89	0.78 ± 0.23	0.75	*p* = 0.001 *
D-P FE	0.88 ± 0.22	0.76	0.61 ± 0.18	0.63	*p* = 0.005 *
P PE-P FE	0.65 ± 0.25	0.62	0.72 ± 0.17	0.74	*p* < 0.001 *
P FE-P FE	0.59 ± 0.15	0.58	0.80 ± 0.16	0.78	*p* < 0.001 *

D—deciduous tooth, P—permanent tooth, PE—partially erupted, FE—fully erupted, SD—standard deviation, Me—median, * statistical significance.

**Table 3:** The width of attached gingiva changes including a type of tooth exchange and degree of eruption.

Exchange/Eruption Degree	Examination I		Examination II		*p* Value
	Mean ± SD	Me	Mean ± SD	Me	
D-P PE	3.80 ± 0.85	4	2.96 ± 1.17	3	*p* < 0.001 *
D-P FE	4.36 ± 1.03	4	2.82 ± 0.98	3	*p* = 0.007 *
P PE-P FE	3.17 ± 0.98	3	2.53 ± 0.93	2.5	*p* < 0.001 *
P FE-P FE	2.75 ± 1.09	2.5	2.60 ± 0.88	2	*p* = 0.372

D—deciduous tooth, P—permanent tooth, PE—partially erupted, FE—fully erupted, SD—standard deviation, Me—median, * statistical significance.

## Data Availability

All the raw data are available from corresponding authors upon reasonable request.
